# Exponential Damping: The Key to Successful Containment of COVID-19

**DOI:** 10.3389/fpubh.2020.580619

**Published:** 2021-01-08

**Authors:** Feng Zhang, Jinmei Zhang, Menglan Cao, Yong Zhang, Cang Hui

**Affiliations:** ^1^Anhui Province Key Laboratory of Wetland Ecosystem Protection and Restoration, School of Resources and Environmental Engineering, Anhui University, Hefei, China; ^2^Xiguan Community Health Center of Liangzhou, Wuwei, China; ^3^Department of Mathematical Sciences, Centre for Invasion Biology, Stellenbosch University, Matieland, South Africa; ^4^Mathematical BioSciences Unit, African Institute for Mathematical Sciences, Cape Town, South Africa; ^5^International Initiative for Theoretical Ecology, Unit 10, London, United Kingdom

**Keywords:** epidemiology, transmission rate, COVID-19, SARS-CoV-2, ecological model, global ranking

## Abstract

Due to its excessive capacity for human-to-human transmission, the 2019 coronavirus disease (COVID-19) has now been declared a global public health emergency. Here we propose a simple model based on exponential infectious growth, but with a time-varying, largely damping, transmission rate. This model provides an excellent fit to the existing data for 46 countries with 10,000+ cases by 16 May 2020, five continents and the entire world. Hence, the model has largely captured the transmission patterns of the COVID-19 outbreak under a variety of intervention and control measures. The damping rate ranged from −0.0228 to 0.1669 d^−1^ globally (a negative damping rate represents acceleration in spread) and can greatly affect the duration of the outbreak and the eventual number of infections. Our model suggests that it is possible to defeat the COVID-19 pandemic by the end of 2020 through achieving a high damping rate (0.0615 d^−1^). However, the global damping rate is rather low (0.0504 d^−1^ before 26 April) and has dropped even further since late April (0.0168 d^−1^). Easing currently implemented control measures in countries with weak or no damping in transmission could lead to an exponential rebound of COVID-19 spread.

## Introduction

The 2019 novel coronavirus (COVID-19), which can cause acute pneumonia, was first reported in Wuhan in December 2019, the capital of Hubei Province in central China (1, 2). Due to the excessively high rate of human-to-human transmission, the virus has quickly spread across all provinces of China and all countries of the world (3). In order to contain this outbreak, governments and healthcare authorities across the globe have taken a series of strict public health measures. Wuhan and all major cities in Hubei, for instance, were sealed off, human movement and traffic prohibited, quarantine imposed on all potentially exposed people, makeshift hospitals quickly built to receive and cure for infected patients. After implementation for just 1 month, these control measures effectively contained the spread of this highly infectious novel coronavirus in China (4), and were considered therefore highly efficient by the World Health Organization (WHO) (5). As the first wave of the pandemic has passed beyond China, COVID-19 now begins to rage worldwide, sweeping across all continents except Antarctica (6). For effective monitoring and containment of the pandemic, it is crucial to understand the patterns of its rapidly changing and localized transmission and promptly evaluate whether the currently implemented control measures are adequate to “flatten the curve.”

Traditional epidemiological models, such as the SIR and SEIR models, explain the rapid increase in the number of infections by the presence of a large susceptible population exposed to infection, and the decline of infection by the gradual depletion of the susceptible population (7). Such a complex model structure is not necessary for capturing the spread of COVID-19 due to the massive size of regional and global susceptible populations (easily running into tens or hundreds of millions of residents in a region). The relatively limited infection, albeit excessively high when focused solely on the sheer number of infections, as well as the resultant mortality, have rather small effects on the demography of regional and global populations, unless a large fraction of the population eventually contracts the virus. In addition, the parameterisation of such models is also unreliable for a novel virus where its pathology and transmission pathways remain unclear with little data support. As such, we here propose a population ecology model with a time-varying infection rate to capture the transmission patterns of COVID-19. The advantage of this phenomenological model is that it does not rely on detailed pathology, yet can still provide an accurate and rapid assessment of COVID-19 transmission patterns under implemented control measures. The rate of exponential damping in transmission rate, as will be shown, provides a real-time evaluation of the efficacy of any implemented control measures.

## Models

Assuming the population is large yet the outbreak limited, so that the impact of infection on the demographic dynamics of the susceptible population is negligible, we could capture the number of infected cases *N*(*t*) over time using an ordinary differential equation,

$$\begin{array}{l} dN\left(t\right)/dt=r\left(t\right)N\left(t\right)\left(1-N\left(t\right)/K\right), \end{array}$$

where *r*(*t*) is the time-dependent transmission rate and *K* the carrying capacity of the number of infections (set as 70% of the entire population; note, in most cases the final number of infections is much lower than *K*, so we have essentially ignored its effect on the outbreak; results are insensitive to changes in K, see Supplementary Figure 1). Notably, *dr*(*t*)/*dt* > 0 represents the acceleration of the viral spread, while *dr*(*t*)/*dt* < 0 indicates the deceleration and damping dynamics.

We define the damping rate (*a*) as the rate of the exponential decline in the transmission rate *r*(*t*); that is, *r*(*t*) = *e*^−*at*+*b*^. An effective control measure should, arguably, result in the deceleration of the spread at a high damping rate (large positive *a*), while inadequate control measures could lead to a low damping rate (small positive *a* close to zero) and even the acceleration of the spread (*a* < 0). The solution to the above differential equation is

$$N\left(t\right)=KN_{0}e^{\int_{t_{0}}^{t}r\left(t\right)dt}/\left(K-N_{0}+N_{0}e^{\int_{t_{0}}^{t}r\left(t\;\right)dt}\right),$$

where *N*_0_ is the number of infected cases at the initial time (*t*_0_), in particular $N(t)\approx N_{0}e^{\int_{t_{0}}^{t}r\left(t\right)dt}$ when ignoring the effect of *K*. Thus, to contain the virus outbreak with any measures, it is necessary to ensure the convergence of $\int_{t_{0}}^{t}r\left(t\right)dt$.

We used the reported data from the WHO to estimate the transmission rate of each day as *r*(*t* + 1/2) = ln(*N*(*t* + 1)) − ln(*N*(*t*)), where *t* is measured in days, and then fitted for parameter *a* and *b*. We estimated the outbreak duration for a region, in a strict sense, as the number of days from a specific date till when the number of daily new infections has dropped to below 0.1 on average, and calculated the corresponding final number of infections. In this simple model, the transmission pattern of an outbreak can be captured solely by the time-varying transmission rate *r*(*t*) itself, which reflects the compound effects of the natural transmission rate under implemented control measures.

## Results

**Figure 1:**
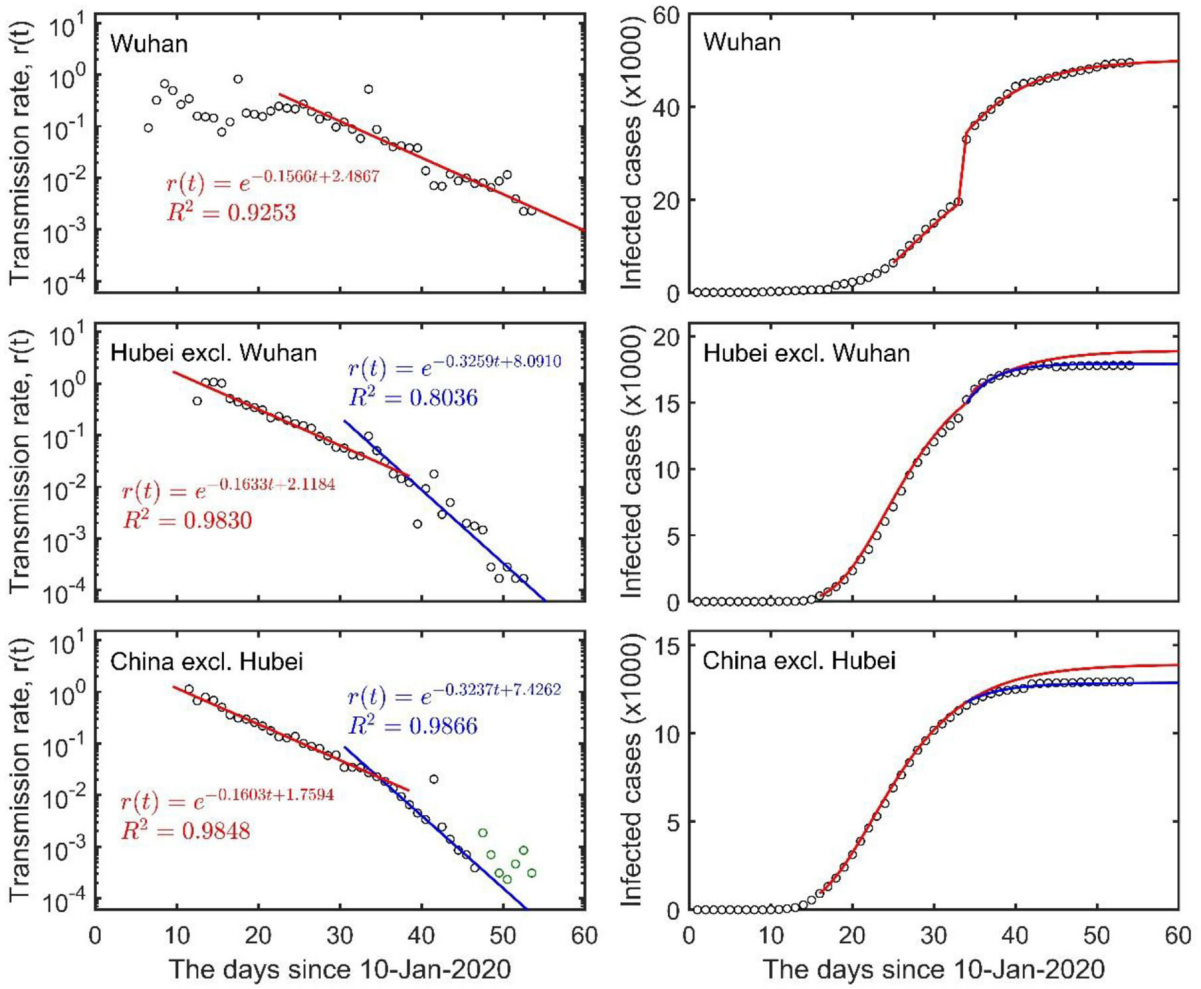
The exponential damping of COVID-19 transmission rates in Wuhan city, Hubei Province (excluding Wuhan) and the rest of Chinese mainland (excluding Hubei) (left column), and the total number of infection cases (right column). The red and blue lines on the left panels represent regressions of the data, and the lines on the right panels are the corresponding predictions using the fitted time-dependent transmission rate [*r* (*t*)]. Circles indicate real data, and green circles indicate cases imported from other countries but were not considered in the regression.

To illustrate our model, we first compiled the daily numbers of COVID-19 infections from the website of the NHCC (www.nhc.gov.cn) for the period of 10 January to 3 March 2020 in Wuhan city, Hubei Province, and the whole of Chinese mainland (consistent with the data in the website of WHO, www.who.int, except for the time difference of 1 day). Evidently, our model provided an excellent fit to the data, unveiling a clear pattern of COVID-19 transmission (Figure 1). The transmission rates in Wuhan, Hubei (but excluding Wuhan), and the rest of Chinese mainland outside Hubei, all began to decline exponentially at around the same damping rate (about 0.16 d^−1^) after the large-scale control measures implemented by the Chinese authorities from 23 January (red lines in the left panels of Figure 1). Exponential damping was more obvious outside Wuhan after 12 February (at a rate of 0.32 d^−1^; see the blue lines in the left panels of Figure 1). Such exponentially damping patterns have accurately captured the spreading dynamics of COVID-19 in China (see right panels of Figure 1), and thus could be considered a reliable monitoring indicator of the effectiveness of those control measures implemented in other global regions for controlling the COVID-19 outbreak.

**Figure 2:**
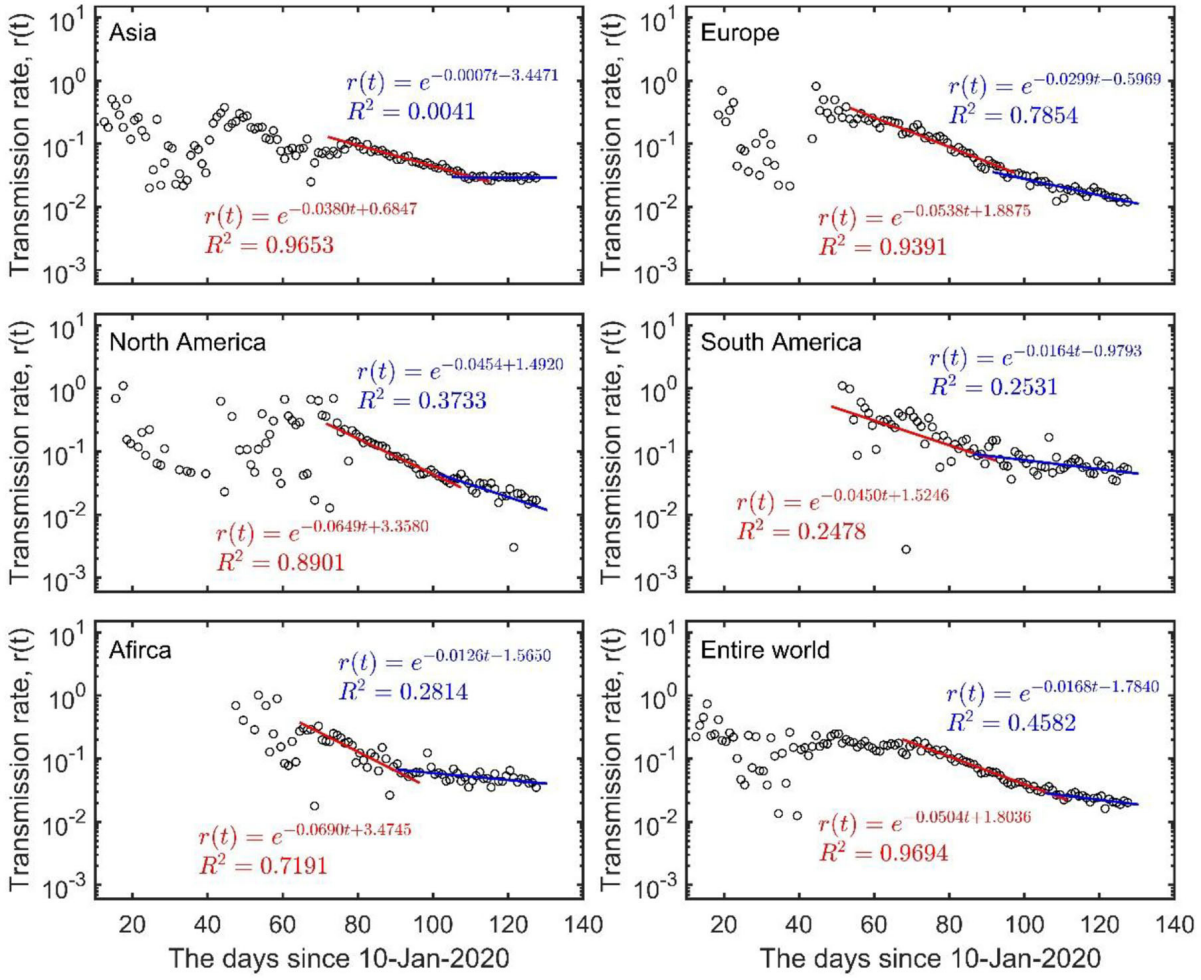
The two-stage damping of COVID-19 in Asia (excl. China), Europe, North America, South America, Africa, and the entire world. The red and blue lines represent the regressions of the data in first and second stage separated at 28 April in Asia, 12 April in Europe, 22 April in North America, 7 April in South America, 11 April in Africa and 26 April for the entire world. Circles indicate the data. See detail in Table 1.

Using the daily infection numbers from 20 January to 16 May 2020, from the WHO website (www.who.int), we analyzed the dynamics of the COVID-19 outbreak in five continents (Asia, Europe, North America, South America, and Africa) and at the global scale. We found that, at the early stage (before 28 April in Asia, 12 April in Europe, 22 April in North America, 7 April in South America and 11 April in Africa), the transmission rates of COVID-19 in all five continents were declining exponentially albeit at a low rate (from 0.038 to 0.069 d^−1^ in Figure 2, compared to 0.16 d^−1^ in mainland China in Figure 1). The damping rates have dropped to a much lower level after these dates (from 0.0007 to 0.0454 d^−1^) when many countries started to ease the lockdown restrictions, with Asia showing no signs of exponential damping (Figure 2 and Table 1). Such a two-stage exponential damping pattern was also evident at the entire global scale, where the damping rate dropped from the initial 0.0504 d^−1^ before 26 April to 0.0168 d^−1^ afterward (Figure 2 and Table 1). Using the current exponential damping rate of 0.0168 d^−1^, we estimated that the COVID-19 could still last for 3 years globally (863 days from 16 May; 95% CI: 532 to 2,110 days), with a total number of 13.9 million infections (95% CI: 8.6–69.1 million). However, if the damping rate were kept at the first-stage level of 0.0504 d^−1^, the global pandemic would only remain for <1 year (258 days from 16 May; 95% CI: 240–278 days), with a total number of 4.6 million infections (95% CI: 4.3–4.9 million). Importantly, it is possible to defeat the COVID-19 pandemic by the end of 2020 through implementing heightened control measures from now on (16 May 2020) to maintain the global damping rate at 0.0615 d^−1^. Evidently, the current changes in damping rate could greatly prolong the duration of the pandemic and the total number of infections by 3-folds, with Asia now facing an uncertain future of indefinite COVID-19 epidemic (see detail in Table 1).

**Table 1:** Damping rates (DR), outbreak duration (days from 16 May 2020) and the final numbers of COVID-19 infections in five continents and for the entire world.

**Continent**	**Damping rate (DR)**		**Outbreak duration**		**Final number of infections (x10^6^)**	
	**1st stage**	**2nd stage**	**With 1st stage DR**	**With 2nd stage DR**	**With 1st stage DR**	**With 2nd stage DR**
Asia	0.0380 (0.0354, 0.0406)	0.0007 (−0.0051, 0.0065)^*^	311 (287, 339)	N/A	0.89 (0.82, 0.98)	N/A
Europe	0.0538 (0.0493, 0.0584)	0.0299 (0.0243, 0.0355)	217 (194, 245)	389 (317, 497)	1.76 (1.56, 2.05)	2.50 (2.26, 2.89)
North America	0.0648 (0.0562, 0.0735)	0.0454 (0.0194, 0.0715)	178 (151, 214)	252 (145, 672)	1.41 (1.25, 1.66)	2.05 (1.75, 3.95)
South America	0.0450 (0.0185, 0.0715)	0.0164 (0.0070, 0.0257)	224 (104, 932)	873 (482, 2,857)	0.16 (0.05, 46.40)	6.40 (1.66, *K*)
Africa	0.0690 (0.0504, 0.0877)	0.0126 (0.0054, 0.0199)	104 (69, 170)	1051 (575, 3,576)	0.03 (0.02, 0.05)	2.11 (0.49, 531.79)
Entire world	0.0504 (0.0474, 0.0534)	0.0168 (0.0078, 0.0258)	258 (240, 278)	863 (532, 2,110)	4.59 (4.34, 4.91)	13.90 (8.63, 69.14)

*An asterisk denotes a non-significant difference from zero damping, and K the maximum possible number of infections; parentheses indicate 95% confidence intervals; N/A represents an unpredictable outcome due to the lack of significant exponential damping*.

**Figure 3:**
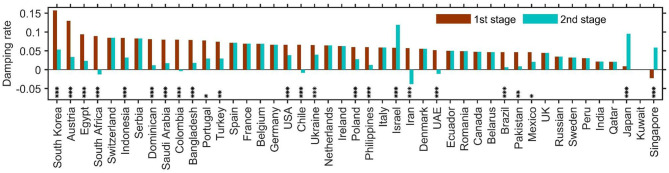
The damping rates of COVID-19 transmission for 45 countries with >10 thousand infections by 16 May. The two stages were defined as before and after late April on average. The countries are ranked according to their first-stage damping rates (see detail in Table 2). Asterisks denote significant difference in damping rate between the two stages: ***p < 0.001; **p < 0.005; *p < 0.05.

We further calculated the damping rate for 46 countries that have reported more than 10 thousand cases by 16 May, from which we also estimated the duration of the outbreak and the final number of infections (Table 2). The aforementioned two-stage transmission pattern was found in most of the 46 countries. There were 21 countries where the second-stage damping rate was lower than that of the first stage (indicative of reduced control measures), while only 3 countries showed the opposite trend (Israel, Japan and Singapore) (indicating intensified control measures). The damping rate in the rest of the 46 countries remained largely unchanged (Figure 3). The reduction of damping rate has expanded the duration of the outbreak and the eventual number of infections manyfold, with some countries now facing unpredictable outbreaks, notably South Africa, Colombia, Chile, Iran and United Arab Emirates (Figure 3; Table 2).

**Table 2:** Damping rates (DR), outbreak duration (days from 16 May 2020) and the final numbers of COVID-19 infections for 46 countries (each with more than 10 thousand cases reported by 16 May).

**Country**	**Damping rate (DR)**		**Outbreak duration**		**Final number of infections**	
	**1st stage**	**2nd stage**	**With 1st stage DR**	**With 2nd stage DR**	**With 1st stage DR**	**With 2nd stage DR**
China	0.1669 (0.1442, 0.1896)	N/A	N/A	N/A	N/A	N/A
South Korea	0.1574 (0.1394, 0.1753)	0.0532 (0.0414, 0.0650)	−16 (−22, −9)	20 (1, 51)	9,029 (8,897, 9,243)	11,151 (11,100, 11,252)
Austria	0.1298 (0.1161, 0.1435)	0.0336 (0.0137, 0.0535)	25 (18, 33)	145 (74, 435)	15,268 (15,025, 15,616)	17,115 (16,528, 19,925)
Egypt	0.0937 (0.0621, 0.1253)	0.0233 (0.0136, 0.0329)	24 (2, 73)	380 (227, 835)	1,196 (919, 2,182)	54,521 (26,952, 345,532)
South Africa	0.0890 (0.0397, 0.1383)	−0.0128 (−0.0205, −0.0051)	47 (4, 263)	N/A	4,065 (1,926, 128,519)	N/A
Switzerland	0.0845 (0.0798, 0.0892)	0.0845 (0.0798, 0.0892)	59 (53, 65)	59 (53, 65)	30,850 (30,752, 30,979)	30,850 (30,752, 30,979)
Indonesia	0.0843 (0.0687, 0.0999)	0.0323 (0.0231, 0.0415)	52 (33, 82)	238 (165, 380)	4,259 (3,353, 6,326)	34,308 (26,242, 57,751)
Serbia	0.0827 (0.0762, 0.0891)	0.0827 (0.0762, 0.0891)	46 (39, 55)	46 (39, 55)	11,124 (10,980, 11,313)	11,124 (10,980, 11,313)
Dominican	0.0809 (0.0660, 0.0958)	0.0116 (−0.0151, 0.0384)^*^	76 (56, 105)	N/A	8,352 (7,305, 10,590)	N/A
Saudi Arabia	0.0798 (0.0540, 0.1055)	0.0173 (0.0066, 0.0281)	60 (29, 129)	708 (363, 130)	5,133 (3,747, 11,279)	825,882 (197,204, 0)
Colombia	0.0796 (0.0617, 0.0976)	−0.0040 (−0.0115, 0.0035)^*^	65 (42, 104)	N/A	4,845 (3,947, 7,195)	N/A
Bangladesh	0.0790 (0.0606, 0.0973)	0.0180 (0.0082, 0.0278)	101 (72, 151)	672 (377, 2064)	15,382 (10,646, 29,120)	461,866 (123,889, 42,611,662)
Portugal	0.0774 (0.0687, 0.0860)	0.0294 (−0.0168, 0.0756)^*^	89 (75, 106)	N/A	28,700 (27,314, 30,797)	N/A
Turkey	0.0741 (0.0695, 0.0787)	0.0295 (−0.0015, 0.0604)^*^	124 (114, 135)	N/A	154,794 (150,050, 160,780)	N/A
Spain	0.0712 (0.0670, 0.0753)	0.0712 (0.0670, 0.0753)	113 (104, 123)	113 (104, 123)	239,524 (237,462, 242,222)	239,524 (237,462, 242,222)
France	0.0692 (0.0635, 0.0749)	0.0692 (0.0635, 0.0749)	109 (96, 124)	109 (96, 124)	145,303 (143,672, 147,663)	145,303 (143,672, 147,663)
Belgium	0.0685 (0.0637, 0.0732)	0.0685 (0.0637, 0.0732)	104 (95, 116)	104 (95, 116)	58,472 (57,661, 59,532)	58,472 (57,661, 59,532)
Germany	0.0662 (0.0612, 0.0712)	0.0662 (0.0612, 0.0712)	120 (108, 134)	120 (108, 134)	182,380 (180,170, 185,440)	182,380 (180,170, 185,440)
Chile	0.0658 (0.0556, 0.0759)	−0.0085 (−0.0215, 0.0046)^*^	105 (82, 140)	N/A	16,342 (13,251, 22,623)	N/A
USA	0.0658 (0.0571, 0.0744)	0.0385 (0.0268, 0.0501)	173 (147, 208)	305 (223, 464)	1,284,888 (1,148,671, 1,503,493)	2,034,897 (1,782,359, 2,638,508)
Ukraine	0.0653 (0.0516, 0.0789)	0.0397 (0.0317, 0.0477)	112 (81, 164)	200 (157, 268)	13,904 (10,048, 24,610)	32,846 (28,047, 42,040)
Netherlands	0.0646 (0.0618, 0.0675)	0.0646 (0.0618, 0.0675)	107 (100, 114)	107 (100, 114)	46,703 (46,220, 47,289)	46,703 (46,220, 47,289)
Ireland	0.0626 (0.0577, 0.0675)	0.0626 (0.0577, 0.0675)	101 (89, 114)	101 (89, 114)	26,681 (26,042, 27,549)	26,681 (26,042, 27,549)
Poland	0.0600 (0.0483, 0.0718)	0.0278 (0.0187, 0.0368)	118 (86, 168)	283 (196, 468)	15,845 (11,993, 25,495)	34,427 (27,355, 55,234)
Philippines	0.0595 (0.0456, 0.0733)	0.0122 (−0.0054, 0.0299)^*^	110 (77, 167)	N/A	10,547 (8,567, 15,831)	N/A
Italy	0.0586 (0.0567, 0.0606)	0.0586 (0.0567, 0.0606)	143 (137, 150)	143 (137, 150)	237,786 (236,209, 239,587)	237,786 (236,209, 239,587)
Israel	0.0579 (0.0493, 0.0665)	0.1191 (0.0830, 0.1551)	125 (100, 161)	18 (5, 44)	21,963 (18,740, 28,147)	16,752 (16,660, 17,000)
Iran	0.0571 (0.0536, 0.0605)	−0.0382 (−0.0558, −0.0205)	147 (135, 160)	N/A	111,125 (108,264, 114,757)	N/A
Denmark	0.0553 (0.0500, 0.0606)	0.0553 (0.0500, 0.0606)	96 (83, 112)	96 (83, 112)	12,100 (11,818, 12,482)	12,100 (11,818, 12,482)
UAE	0.0515 (0.0484, 0.0547)	−0.0112 (−0.0331, 0.0107)^*^	167 (154, 181)	N/A	28,367 (26,444, 30,776)	N/A
Ecuador	0.0498 (0.0344, 0.0651)	0.0498 (0.0344, 0.0651)	135 (84, 238)	135 (84, 238)	38,247 (33,434, 56,214)	38,247 (33,434, 56,214)
Romania	0.0489 (0.0445, 0.0533)	0.0489 (0.0445, 0.0533)	141 (124, 163)	141 (124, 163)	21,059 (19,894, 22,727)	21,059 (19,894, 22,727)
Canada	0.0472 (0.0416, 0.0528)	0.0472 (0.0416, 0.0528)	180 (153, 214)	180 (153, 214)	99,761 (92,019, 112,012)	99,761 (92,019, 112,012)
Belarus	0.0466 (0.0368, 0.0563)	0.0466 (0.0368, 0.0563)	186 (139, 263)	186 (139, 263)	62,964 (46,924, 105,204)	62,964 (46,924, 105,204)
Brazil	0.0465 (0.0355, 0.0575)	0.0065 (−0.0025, 0.0155)^*^	215 (153, 332)	N/A	124,412 (64,428, 411,746)	N/A
Pakistan	0.0464 (0.0228, 0.0700)	0.0085 (0.0010, 0.0161)	169 (83, 534)	1815 (741, 120)	19,556 (8,739, 424,480)	14,000,045 (585230, *K*)
Mexico	0.0463 (0.0166, 0.0760)	0.0208 (0.0164, 0.0253)	180 (67, 176)	547 (416, 770)	23,002 (4,513, *K*)	437,846 (240,955, 1,130,187)
UK	0.0443 (0.0415, 0.0471)	0.0443 (0.0415, 0.0471)	232 (215, 253)	232 (215, 253)	330,253 (312,990, 352,592)	330,253 (312,990, 352,592)
Russian	0.0345 (0.0308, 0.0382)	0.0345 (0.0308, 0.0382)	345 (300, 404)	345 (300, 404)	974,546 (758,990, 1352,733)	974,546 (758,990, 1,352,733)
Sweden	0.0323 (0.0281, 0.0365)	0.0323 (0.0281, 0.0365)	269 (230, 322)	269 (230, 322)	49,830 (43,939, 59,403)	49,830 (43,939, 59,403)
Peru	0.0305 (0.0240, 0.0369)	0.0305 (0.0240, 0.0369)	359 (272, 506)	359 (272, 506)	325,187 (200,867, 744,550)	325,187 (200,867, 744,550)
India	0.0213 (0.0165, 0.0262)	0.0213 (0.0165, 0.0262)	560 (423, 800)	560 (423, 800)	785,355 (439,619, 2,023,297)	785,355 (439,619, 2,023,297)
Qatar	0.0209 (0.0149, 0.0269)	0.0209 (0.0149, 0.0269)	528 (370, 854)	528 (370, 854)	272,727 (130,918, 1,085,372)	272,727 (130,918, 1,085,372)
Japan	0.0090 (0.0012, 0.0168)	0.0952 (0.0868, 0.1037)	1640 (606, 94)	30 (23, 38)	5,885,400 (97,523, 0)	16,838 (16,696, 17,029)
Kuwait	−0.0003 (−0.0073, 0.0068)^*^	−0.0003 (−0.0073, 0.0068)^*^	N/A	N/A	N/A	N/A
Singapore	−0.0228 (−0.0306, −0.0149)	0.0586 (0.0422, 0.0749)	N/A	128 (89, 199)	N/A	38,611 (33,570, 50,874)

*N/A represents an unpredictable outcome due to the lack of significant exponential damping or the number of daily cases already extremely low (e.g., China), the other symbols are the same as Table 1*.

**Figure 4:**
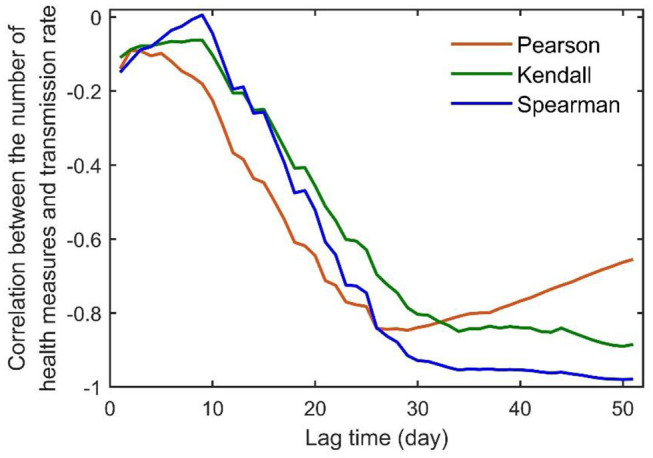
Lag-dependent correlation between the number of additional health measures implemented from January 24 to March 26 and the transmission rate for the entire world. The data on health measures were extracted from “Coronavirus disease 2019 (COVID-19) Situation Report-67” reported by the WHO (8).

Lastly, using the cumulative number of additional health measures reported from 24 January to 26 March 2020 across the world (8), we explored the relationship between the transmission rate of COVID-19 and implemented control measures. We report here a strong negative correlation between the transmission rate to the number of control measures, but with a lag of about 30 days in the transmission rate to respond to the implemented control measures (Figure 4). In addition, the damping rate at the second stage was found to be positively correlated with a country's GDP per capita, average lifespan, doctor and nurse density per 10,000 population, and negatively correlated with the Gini coefficient; however, such correlations do not appear for the damping rate at the first stage (Table 3; data on human health indices from WHO, www.who.int, and OECD, www.oecd.org).

**Table 3:** Spearman correlations between human health indices and the damping rate of COVID-19 transmission.

**Human health index**	**Damping rate**	
	**1st stage**	**2nd stage**
Population	0.1217 ns	−0.2531 ns
GDP	0.02193 ns	0.2779 ns
GDP per capita	−0.1712 ns	0.4867^***^
Average lifespan	0.04602 ns	0.6654^***^
Gini coefficient	−0.04511 ns	−0.6421^*^
Health expenditure per capita	−0.06069 ns	0.5665^***^
Doctor density per 10000	−0.02175 ns	0.63001^***^
Nurse density per 10000	−0.1472 ns	0.47974^**^

Significance levels:

*** p < 0.0001;

** p < 0.001;

* p < 0.005;

*ns, not significant with p > 0.05*.

## Discussion

The data from China show that it is possible to contain the spread of COVID-19; this is signaled by the exponential damping of the transmission rate (Figure 1). Such exponential damping is also evident from the data of the 2003 SARS outbreak in mainland China, Hong Kong and the entire world (data from the website of WHO, Supplementary Figure 2). This implies that exponential damping in disease transmission could be a universal pattern of successful infectious disease containment. The damping rate of virus transmission is strongly related to the outbreak duration in the form of a power law but has only a trivial correlation with the time elapsed since the first locally reported case (Supplementary Figure 3); consequently, the damping rate reflects the effectiveness of implemented control measures over the natural infection rate of the disease. Its variation across countries therefore reveals whether the current implemented local/regional measures are adequate (see Figure 1, Tables 1, 2). Such country-level variations in the damping rate, therefore, also reflect a country's socioeconomics and human health conditions as captured here by the country's GDP per capita, average lifespan, doctor and nurse density, and Gini coefficient (Table 3). By estimating the time-varying transmission rate and its damping rate, our model provides a simple theoretical framework for monitoring the spread of an outbreak and assessing the efficacy of implemented control measures in real time. This is important for regional decision-makers and global governance to reflect upon, in order to modify any implemented control measures and practices in time.

Theoretically, our model can be used for rapid evaluation of the pandemic outbreak in real time and assessment of any intervention measures. To this end, control measures from countries and regions that have already shown exponential damping in their transmission rates could be communicated and compared by the WHO for better local disease control worldwide. If countries were able to maintain the damping rate at 0.16 d^−1^, as shown possible in China, the global COVID-19 pandemic would end in 3 months (from 16 May) with the total number of infections <5 million. However, our analysis suggests that, since late April the pandemic has rebounded to a lower damping rate than before at the global scale, and it is accelerating exponentially at the moment in some countries where the first-stage exponential damping during March to early April has been disrupted. This rebound will drastically prolong the anticipated duration of the outbreak and increase the final number of infections, with a few countries facing extremely uncertain futures. Additional control measures should be implemented in countries showing a low damping rate or no signs of exponential damping. Slight improvement of the current control measures can bring about drastic improvement on outbreak control, especially in countries lacking exponential damping in transmission. Globally, our analysis suggests that it is possible to defeat the COVID-19 pandemic by the end of 2020 only if all countries take immediate control measures to achieve a damping rate of 0.0615 d^−1^ from the current rate of 0.0168 d^−1^. Moreover, it is crucial to implement rapid control measures due to the month-long lag in the transmission rate in respondence to any effective control measures (Figure 4). Seeing the multiple waves of COVID-19 outbreaks in many parts of the world, we hope that the damping rate can be used as an instantaneous index for effective disease controls at both regional and global scales.

## Data Availability Statement

Publicly available datasets were analyzed in this study. This data can be found here: The website of WHO (www.who.int).

## Author Contributions

FZ and CH conceived the idea. JZ, MC, and YZ compiled the data. FZ and CH ran the analyses and wrote the paper. All authors contributed further editing on the final version.

## Conflict of Interest

The authors declare that the research was conducted in the absence of any commercial or financial relationships that could be construed as a potential conflict of interest.
