# Supplementary material for: Exponential Damping: The Key to Successful Containment of COVID-19
Source: Front Public Health. 2021 Jan 8;8:580619. doi: 10.3389/fpubh.2020.580619 (PMC7820870; doi:10.3389/fpubh.2020.580619)
Supplement: Supplementary file 1 [file Data_Sheet_1.docx]

**Appendix S1**: Fig.S1-S3


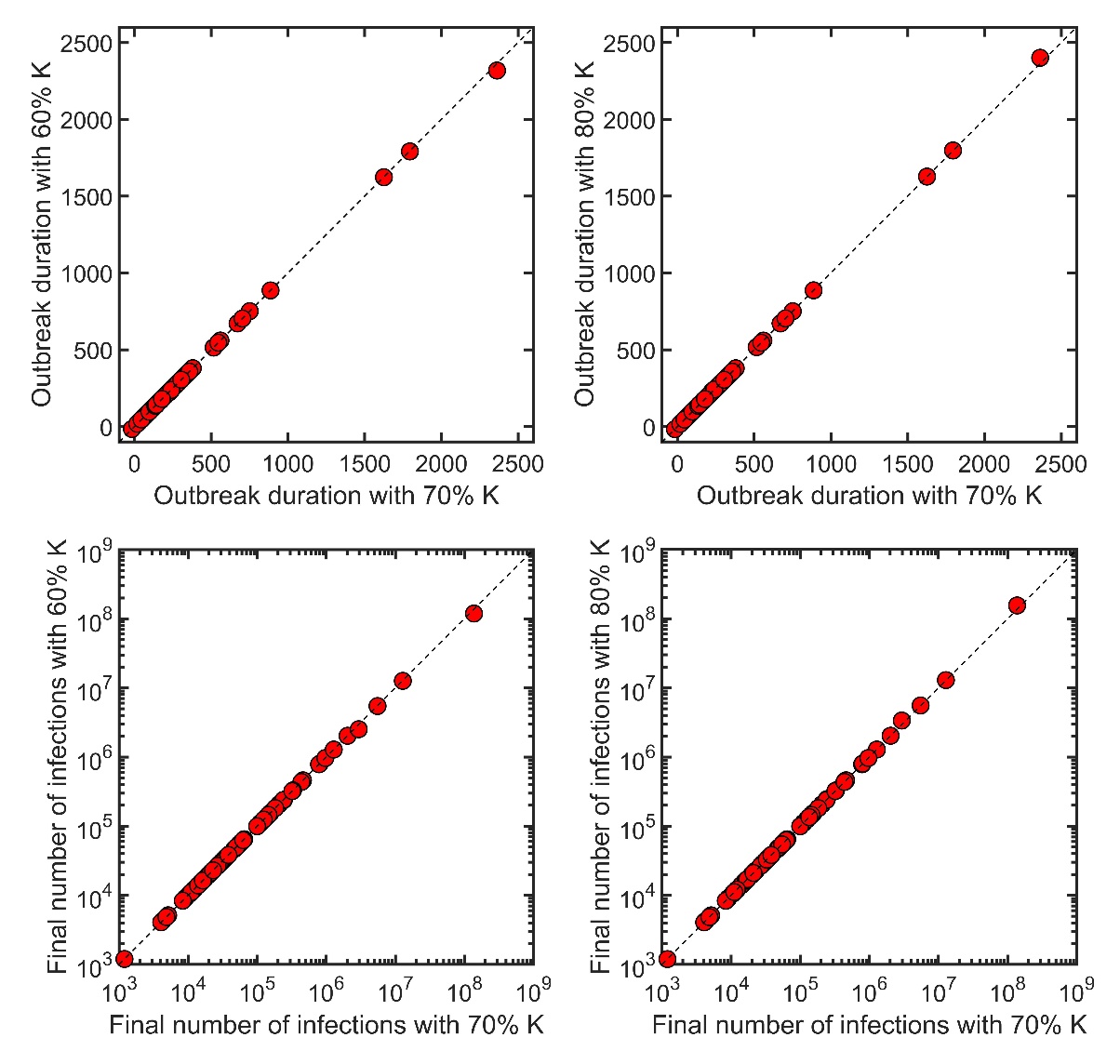


**Fig.S1:** Insensitivity of K to outbreak duration and final number of infections predicted by the model.


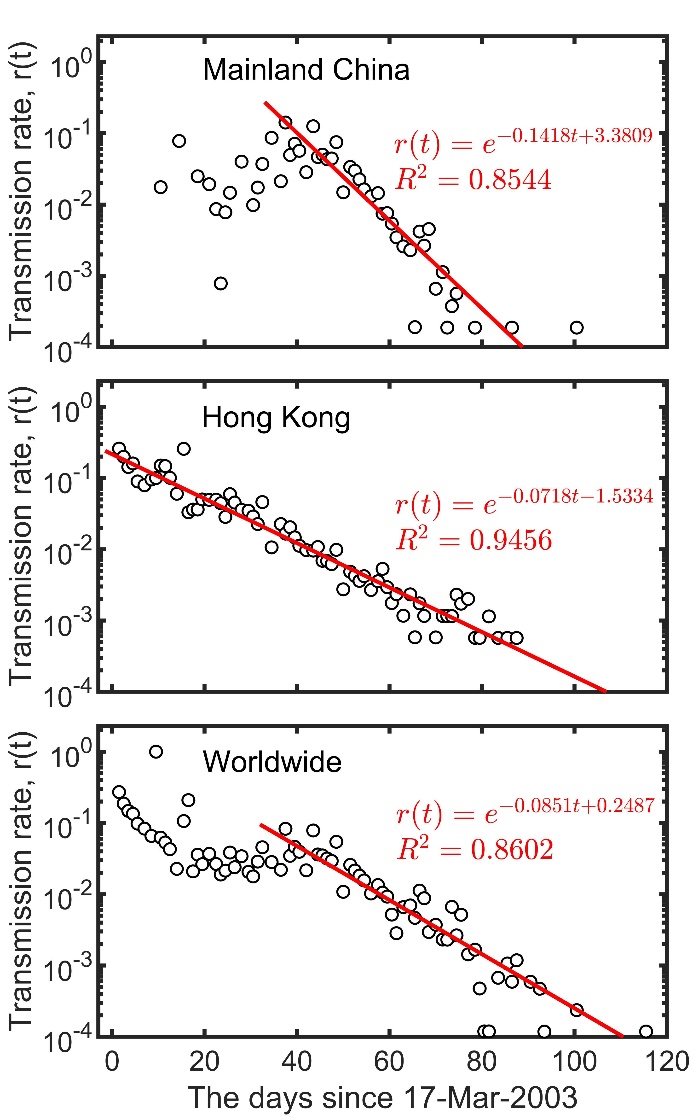


**Fig.S2:** The exponential damping patterns of the 2003 SARS. Data from the website of WHO ([www.who.int](http://www.who.int)).

**
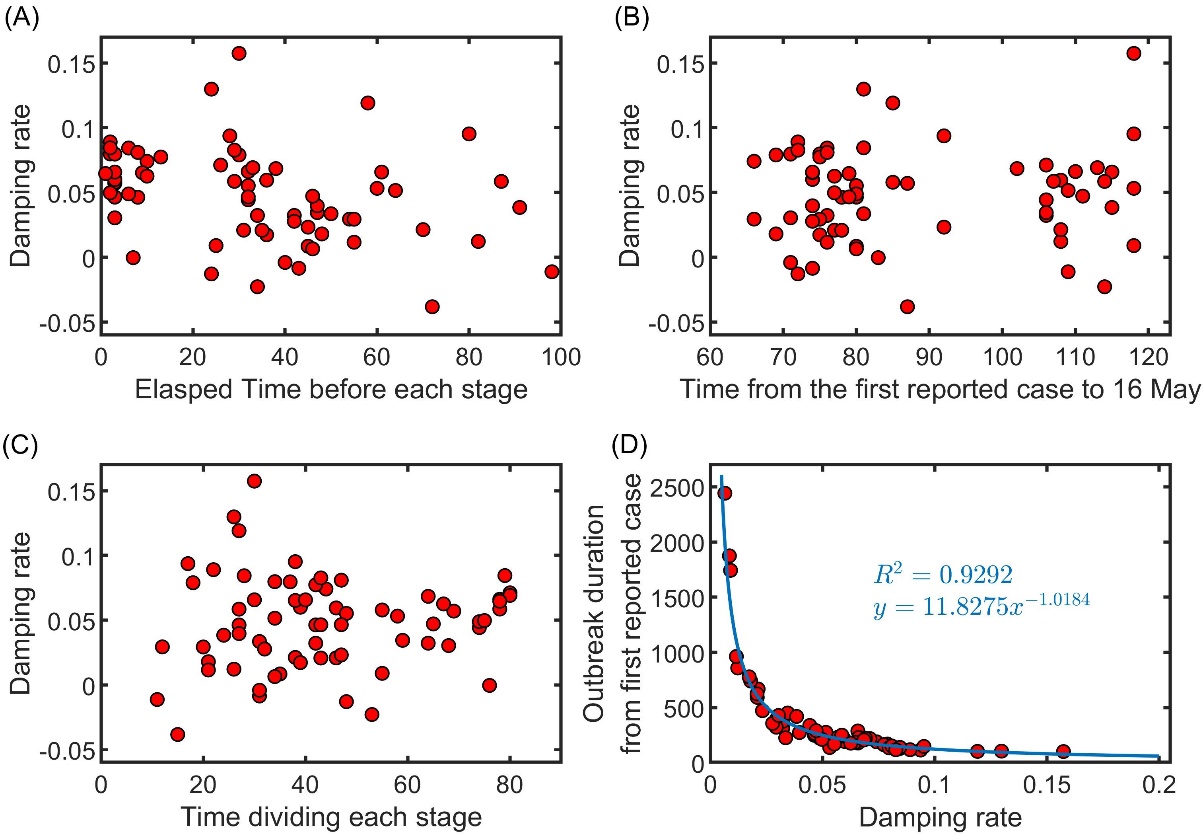
**

**Fig.S3:** Damping rate uncorrelated to the elapsed time before each stage (A), the time from first reported case to 16-May-2020 (B), and the time dividing the first stage and second stage (C), but showing a strong power law relationship with predicted outbreak duration from the day when first case reported (D).

**Appendix S2:** MATLAB Code for calculations

% This code is used to calculate time-varying transmission rate,

% and fit parameter a and b, and make prediction.

% The variable x is assigned with the confirmed cases

% before running the code, and k is the population of the country.

T = (2:length(x))'-0.5;

D = log(x(2:end))-log(x(1:end-1)); % transmission rate

[coef,bint,R,rint,states] = regress(D,[ones(size(T)),T]);

a = coef(2);% -a is damping rate

b = coef(1);

px1 = x(end);

t = length(x);

dt = 0.1;

err = 100;

while err>0.01

r = exp(a*(t+dt/2)+b);

px2 = px1*exp(dt*r*(1-px1/(0.7*k)));

err = abs(px2-px1);

px1 = px2;

t = t+dt;

end

disp([-a,t,px2])

% t is outbreak duration and px2 final number of infections
